# Overdose experiences among injection drug users in Bangkok, Thailand

**DOI:** 10.1186/1477-7517-7-9

**Published:** 2010-05-13

**Authors:** M-J Milloy, Nadia Fairbairn, Kanna Hayashi, Paisan Suwannawong, Karyn Kaplan, Evan Wood, Thomas Kerr

**Affiliations:** 1 School of Population and Public Health, University of British Columbia, 5804 Fairview Avenue, Vancouver, British Columbia, V6T 1C3, Canada; 2 British Columbia Centre for Excellence in HIV/AIDS, St. Paul's Hospital, 667-1081 Burrard Street, Vancouver, British Columbia, V6Z 1Y6, Canada; 3 Thai AIDS Treatment Action Group, 18/89 Vipawadee Road, soi 40, Chatuchak, Bangkok, Thailand; 4 Department of Medicine, University of British Columbia, Room 10203, 2775 Laurel Street, Vancouver, British Columbia, V5Z 1M9, Canada

## Abstract

**Background:**

Although previous studies have identified high levels of drug-related harm in Thailand, little is known about illicit drug overdose experiences among Thai drug users. We sought to investigate non-fatal overdose experiences and responses to overdose among a community-recruited sample of injection drug users (IDU) in Bangkok, Thailand.

**Methods:**

Data for these analyses came from IDU participating in the Mit Sampan Community Research Project. The primary outcome of interest was a self-reported history of non-fatal overdose. We calculated the prevalence of past overdose and estimated its relationship with individual, drug-using, social, and structural factors using multivariate logistic regression. We also assessed the prevalence of ever witnessing an overdose and patterns of response to overdose.

**Results:**

These analyses included 252 individuals; their median age was 36.5 years (IQR: 29.0 - 44.0) and 66 (26.2%) were female. A history of non-fatal overdose was reported by 75 (29.8%) participants. In a multivariate model, reporting a history of overdose was independently associated with a history of incarceration (Adjusted Odds Ratio [AOR] = 3.83, 95% Confidence Interval [CI]: 1.52 - 9.65, *p *= 0.004) and reporting use of drugs in combination (AOR = 2.48, 95% CI: 1.16 - 5.33, *p *= 0.019). A majority (67.9%) reported a history of witnessing an overdose; most reported responding to the most recent overdose using first aid (79.5%).

**Conclusions:**

Experiencing and witnessing an overdose were common in this sample of Thai IDU. These findings support the need for increased provision of evidence-based responses to overdose including peer-based overdose interventions.

## Background

Accidental illicit drug-related overdose is a leading cause of preventable morbidity and mortality. In many settings, fatal overdose is the primary contributor to highly elevated mortality rates among injection drug users (IDU) [1,2]. According to several studies of community-recruited IDU, non-fatal overdose is common and associated with factors including having a prior history of overdose, recent incarceration and higher-intensity forms of drug use, such as poly-drug use [3-6]. Several interventions to lower the incidence or reduce the damaging sequelae of overdose events have been implemented, including treatment for drug use [7], drug substitution therapy [8], supervised injection facilities [9] and peer-driven responses, such as naloxone distribution [10].

Despite reports of injection drug use from all major regions of the world [11,12], the phenomenon of accidental drug overdose has not been well described outside of Western settings. In northern Vietnam, over 80% of out-of-treatment male opiate injectors reported a history of overdose in a cross-sectional survey [13]. Overdose in the previous 12 months was common among 731 IDU in Sichuan province, China, and associated with daily heroin use and an injection career of at least seven years in duration [14].

In Thailand, some aspects of drug-related harm, including high levels of incarceration [15], persecution by police [16] and infection with HIV [17,18] hepatitis C [19] and other pathogens [20] have been identified among the estimated 20,000 - 160,000 IDU in the country [11,12]. However, we are unaware of any study that analyses the phenomenon of overdose among Thai drug users. Thus, we sought to estimate the prevalence and correlates of non-fatal overdose, as well as investigate patterns of response to overdose in a community-recruited sample of active IDU in Bangkok, Thailand.

## Methods

Data for these analyses was obtained from the Mit Sampan Community Research Project (MSCRP), a collaborative research effort involving the Mit Sampan Harm Reduction Center (Bangkok, Thailand), the Thai AIDS Treatment Action Group (Bangkok, Thailand), Chulalongkorn University (Bangkok, Thailand) and the British Columbia Centre for Excellence in HIV/AIDS (Vancouver, Canada). In 2008, the research partners designed and undertook a cross-sectional epidemiological study of IDU recruited through peer-based outreach and word-of-mouth. Invited participants were asked to attend the Mit Sampan Harm Reduction Center to be included in the study. All participants provided informed consent and completed an interviewer-administered questionnaire. The survey instrument elicited demographic data, information about past and current drug use, HIV risk behaviour, overdose experiences, interactions with the criminal justice system including police forces and incarceration, and experience with health care. Upon completion of the questionnaire participants were provided a stipend of 250 Thai baht. The study was approved by the research ethics boards at the University of British Columbia and Chulalongkorn University.

For these analyses, the primary endpoint of interest was reporting a history of non-fatal overdose by answering "Yes" to the question: "Have you ever overdosed by accident (i.e., a period of loss of consciousness or breathing?)" In follow-up questions, individuals reporting a history of non-fatal overdose were also asked the type of drug or drugs they were using at the time of their last overdose, if they were helped, and by who, during their last overdose.

As a first step, we investigated the characteristics of individuals with a history of overdose. Explanatory variables included: Age; gender (male vs. female); education level (<*prathom suksa *[elementary-level] vs. ≥ *prathom suksa*); reporting any income from illegal sources (yes vs. no); participation in the sex trade (yes vs. no); history of heroin injection (yes vs. no); history of Midazolam (a benzodiazepine) injection (yes vs. no); history of *yaba *(methamphetamine and caffeine) injection (yes vs. no); history of ice (methamphetamine) injection (yes vs. no); history of using drugs in combination (yes vs. no); history of methadone injection (yes vs. no); ever using an unsterile syringe (yes vs. no); ever lending syringes (yes vs. no); ever incarcerated (yes vs. no); ever on methadone maintenance therapy (MMT) (yes vs. no); and ever in forced drug treatment (yes vs. no). Pearson's X^2^-test and Fisher's exact test were used to determine bivariate relationships.

Next, we used an *a priori*-defined statistical protocol based on examination of the Akaike Information Criterion (AIC) and *p*-values to construct an explanatory multivariate logistic regression model. First, we constructed a full model including all variables analysed in bivariate analyses. After noting the AIC of the model, we removed the variable with the largest *p*-value and built a reduced model. We continued this iterative process until no variables remained for inclusion. We selected the multivariate model with the lowest AIC score.

In a secondary analysis, all participants were asked if they had ever witnessed an overdose. Those with a history of witnessing overdose were asked about their response to the most recently witnessed overdose. Finally, all participants were asked if they believed they had enough information to prevent and manage overdose and what steps they believe should be taken to effectively manage overdose.

## Results

Two-hundred fifty-two individuals were recruited and included in these analyses, of whom 66 (26.1%) were women. The median age at time of interview was 36.5 years (IQR: 29.0 - 44.0 years.) In total, 75 participants (29.8%) reported a history of non-fatal overdose. When asked about the type and routes of administration of all drugs consumed prior to their last overdose, almost all (70, 93.3%) reported injection heroin, followed by injection Midazolam (24, 32.0%), non-injection heroin (11, 14.7%) and non-injection midazolam (4, 5.3%). No other response (including injection and non-injection *yaba*, non-injection ecstasy, injection and non-injection methadone, injection and non-injection benzodiazepine and injection and non-injection alcohol) exceeded three (4.0%) reports.

Of the 75 participants with a history of overdose, 59 (78.7%) reported being helped by another individual during their last overdose. Most reported being assisted by a friend (46, 78.0%), relative (11, 18.6%) or sex partner (3, 5.1%). Of all individuals reporting an overdose, only 28 (33.5%) reported being seen by a healthcare professional.

Results of the univariate analyses of factors associated with reporting a history of non-fatal overdose are presented in Table 1. As shown, the outcome was associated at the *p *< 0.05 level with: reporting a history of incarceration (Odds Ratio [OR] = 4.40, 95% Confidence Interval [CI]: 1.80 - 10.79); a history of using drugs in combination (OR = 3.05, 95% CI: 1.53 - 6.07); and a history of injecting Midazolam (OR = 2.20, 95% CI: 1.67 - 4.12). A history of injecting heroin was significantly associated with reporting ever experiencing a non-fatal overdose (*p *= 0.002); however, as all individuals with a history of overdose also reported a history of heroin injection, an Odds Ratio could not be calculated and that explanatory factor was removed from further consideration. The final multivariate model, presented in Table 2, included two factors independently associated with the outcome: Ever using drugs in combination (Adjusted Odds Ratio [AOR] = 2.48, 95% CI: 1.16 - 5.33) and reporting a history of incarceration (AOR = 3.83, 95% CI: 1.52 - 9.65).

**Table 1:** Univariate analyses of factors associated with reporting a history of non-fatal overdose among IDU in MSHRC cohort (n = 252 individuals).

Characteristic	History of overdose n (%)		OR^1^	95% CI^2^	p-val
				
	*No: 177 (70.2)*	*Yes: 75 (29.8)*			
AGE					

Median (IQR)	37.0 (29.5 - 44.5)	35.0 (28.0 - 42.0)	0.99	0.97 - 1.03	0.843

GENDER					

Male	130 (73.4)	56 (74.7)			

Female	47 (26.6)	19 (25.3)	0.93	0.51 - 1.74	0.877

EDUCATION					

≥ Secondary	110 (62.1)	50 (66.7)	1.00		

< Secondary	37 (37.9)	25 (33.4)	0.82	0.47 - 1.45	0.568

SEX TRADE					

No	167 (94.4)	71 (94.7)	1.00		

Yes	10 (5.6)	4 (5.3)	0.94	0.29 - 3.10	0.841

EVER INJECT HEROIN					

No	18 (10.1)	0 (0.0)			

Yes	159 (89.9)	75 (100.0			0.002

EVER INJECT YABA					

No	66 (37.3)	25 (33.3)	1.00		

Yes	111 (62.7)	50 (66.7)	1.19	0.67 - 2.10	0.570

EVER INJECT MIDAZOLAM					

No	66 (37.3)	16 (21.3)	1.00		

Yes	111 (62.7)	59 (78.7)	2.20	1.67 - 4.12	0.018

EVER INJECT BENZODIAZEPINES					

No	174 (98.3)	73 (97.3)	1.00		

Yes	3 (1.7)	2 (2.7)	1.59	0.26 - 9.71	0.636

EVER INJECT METHADONE					

No	150 (84.7)	63 (84.0)	1.00		

Yes	27 (15.3)	12 (16.0)	1.06	0.50 - 2.22	0.851

EVER USE DRUGS IN COMBINATION					

No	65 (36.7)	12 (16.0)	1.00		

Yes	112 (63.3)	63 (84.0)	3.05	1.53 - 6.07	< 0.001

EVER INCARCERATED					

No	49 (27.7)	6 (8.0)	1.00		

Yes	128 (72.3)	69 (92.0)	4.40	1.80 - 10.79	< 0.001

EVER ON MMT					

No	102 (57.7)	39 (52.0)	1.00		

Yes	75 (42.3)	36 (48.0)	1.26	0.73 - 2.16	0.488

EVER IN FORCED DRUG TREATMENT					

No	127 (71.8)	45 (60.0)	1.00		

Yes	50 (28.2)	30 (40.0)	1.69	0.96 - 2.82	0.076

1. Odds Ratio; 2. 95% Confidence Interval

**Table 2:** Multivariate logistic regression analysis of factors associated with reporting a history of non-fatal overdose in MSHRC cohort (*n *= 252 individuals).

Characteristic	AOR^1^	95% CI^2^	*p*-value
Ever injected Midazolam (Yes vs. no)	1.38	0.68 - 2.81	0.379

Ever used in combination (Yes vs. no)	2.48	1.16 - 5.33	0.020

Ever incarcerated (Yes vs. no)	3.83	1.52 - 9.65	0.004

Ever in forced treatment (Yes vs. no)	1.25	0.69 - 2.28	0.457

1. Adjusted Odds Ratio; 2. 95% Confidence Interval

Experience witnessing an overdose was reported by 171 (67.9%) participants. When asked their response to the last overdose witnessed, most (136, 79.5%) reported performing first aid; 78 (45.6%) took the overdose sufferer to a hospital; 4 (2.3%) took them to the Mit Sampan Harm Reduction Centre; 1 (0.6%) contacted the police. Twelve individuals (7.0%) reported they did nothing in response.

Approximately half of the participants reported they believed they had enough information to prevent (139, 55.2%) and manage (128, 50.8%) an overdose. When asked how to manage an overdose, responses were: perform first aid (115, 45.6%); inject salt water (109, 43.2%); perform CPR (90, 35.7%); slap (105, 41.7%); administer naloxone (16, 6.3%); or take to a hospital (74, 29.4%).

## Discussion

In these analyses, we found a history of non-fatal overdose was common among Thai IDU, with more than one-quarter of the sample (29.8%) reporting a previous overdose event. The predominant drug implicated in overdose events was heroin, with the majority of individuals reporting injecting heroin before their last overdose and every individual with a history of overdose also reporting a history of heroin use. In a multivariate model, a history of overdose was linked to poly-drug use and incarceration. Most of the participants also reported experience witnessing an overdose (67.9%) and the most common responses included performing first aid and taking the victim to a hospital. When asked how to manage an overdose, the most common responses included performing first aid or artificial respiration and injecting salt water.

The level of non-fatal overdose observed in this sample is on the lower end of the range of estimates calculated in similar studies of community-based IDU in Baltimore, Maryland (24.7%) [21]; London, England (37.8%) [22] and San Francisco, California (47.9%) [23]. We are unable to determine if this comparatively lower level is the result of a lower incidence of overdose among Thai IDU or a greater risk of death at each overdose event. Several points of evidence support a contribution from the latter effect, including the high prevalence of witnessing overdoses; the pervasive level of misperceptions concerning how to manage an overdose; the high prevalence of overdose as the reported cause of death among Thai IDU in two HIV vaccine preparatory studies [24,25]; and the ongoing violent crackdown by Thai police against drug users, a phenomenon linked to a greater risk of overdose mortality in other settings [26-28].

Our findings identify the need for enhanced education for Thai IDU to prevent and manage overdoses. Specifically, approximately half of respondents indicated they did not have the information required to prevent and manage overdoses. This lack of knowledge was reflected in the substantial proportion of participants reporting inappropriate responses, including injecting the sufferer with salt water. Given that witnessing an overdose was common in this setting and fatal overdoses typically take hours to develop [29,30], the need to improve peer responses is clear. Inappropriate or suboptimal responses by IDU to overdose are not uncommon and have been reported from a number of settings [26,29,31]. However, overdose management education has been shown to be effective at training IDU to respond appropriately to overdose [26,32].

These findings also support the distribution of naloxone to drug users. Naloxone, an opiate antagonist, is the standard treatment used by healthcare professionals in resuscitation efforts following opioid overdose. Programs to train IDU in overdose response alongside distribution of naloxone would likely benefit Thai IDU, given that opiates were the most common class of drugs reported by this sample prior to their last overdose. Additionally, given pervasive anti-drug user stigma [33,34] and the ongoing violent campaign by police [35], many IDU may be unwilling to seek professional health care in the event of an overdose. Evaluations of analagous interventions in Chicago [36], New York City [10] and San Francisco [37] have observed positive impacts, including hundreds of successful peer opioid overdose resuscitations. Currently, naloxone is only available to IDU in Thailand at the MSHRC.

In the multivariate model, a history of incarceration was independently associated with ever overdosing. This is in line with previous analyses that have identified a high risk of overdose, including fatal overdose, associated with incarceration, especially in the first weeks following release from detention [38,39]. In the Thai context, previous studies have described the links between exposure to correctional environments and an elevated risk of HIV infection among IDU [40,41]. Our findings add evidence supporting the need for an expansion of harm reduction opportunities in Thai correctional settings, such as substitution therapies, shown effective at reducing HIV risk behaviours [42] and improving outcomes post-release [43].

While the implementation of peer-based interventions might lower the incidence and severity of overdose events among Thai IDU, our findings also have implications for other social- and structural-level policies. In particular, our findings are another example of how the reliance on enforcement-based strategies to respond to illicit drug use can produce further drug-related harms [44,45]. Just as some observers have identified deaths resulting from the Thai government's crackdown on drug users [35], our findings describe how criminal justice interventions can increase the risks associated with overdose events. We echo other authors who have credited the country's successful efforts to reduce the incidence of sexually-transmitted HIV infections to the government's adoption of evidence-based policies [41,46] and urge a similar pragmatic initiative to replace dominant enforcement- and suppression-based policies with harm reduction programmes.

Our study has limitations. First, cross-sectional analyses are unable to determine the temporal relationship between outcome and exposure. Second, although our measures are based on self-reports from IDU, we do not believe participants would have been more or less likely to report a history of overdose based on the covariates we examined. Finally, our sample of IDU was not recruited at random and thus may not necessarily generalize to other samples of IDU in Thailand or other settings.

## Conclusions

We observed that non-fatal overdose events were common in this sample of Thai IDU. In a multivariate analysis, reporting a history of non-fatal overdose was independently associated with ever being incarcerated and ever using drugs in combination. A majority of participants reported witnessing overdoses as well as needing more information to respond appropriately. Our findings support the need to expand appropriate harm reduction strategies for drug users in Thailand, such as peer-based overdose management including naloxone distribution, and further highlight the need to balance the current emphasis on enforcement-based responses to illicit drug use with health-focused interventions.

## Competing interests

The authors declare that they have no competing interests.

## Authors' contributions

TK, EW, PS and KK conceived and designed the study; KK, PS, NF and KH implemented the study design, including data acquisition; M-JM performed the statistical analysis, wrote the manuscript and coordinated all revisions; all authors revised the manuscript and read and approved the final draft.
